# The Effectiveness of Screening for Diabetes and Cardiovascular Disease Risk Factors in a Community Pharmacy Setting

**DOI:** 10.1371/journal.pone.0091157

**Published:** 2014-04-01

**Authors:** Andrew Willis, Peter Rivers, Laura J. Gray, Melanie Davies, Kamlesh Khunti

**Affiliations:** 1 Diabetes Research Unit, University of Leicester, Leicester, United Kingdom; 2 DeMontford University, Leicester, United Kingdom; German Diabetes Center, Leibniz Center for Diabetes Research at Heinrich Heine University Duesseldorf, Germany

## Abstract

Risk factors for cardiovascular disease including diabetes have seen a large rise in prevalence in recent years. This has prompted interest in prevention through the identifying individuals at risk of both diabetes and cardiovascular disease and has seen increased investment in screening interventions taking place in primary care. Community pharmacies have become increasingly involved in the provision of such interventions and this systematic review and meta-analysis aims to gather and analyse the existing literature assessing community pharmacy based screening for risk factors for diabetes and those with a high cardiovascular disease risk.

**Methods:**

We conducted systematic searches of electronic databases using MeSH and free text terms from 1950 to March 2012. For our analysis two outcomes were assessed. They were the percentage of those screened who were referred for further assessment by primary care and the uptake of this referral.

**Results:**

Sixteen studies fulfilled our inclusion criteria comprising 108,414 participants screened. There was significant heterogeneity for all included outcomes. Consequently we have not presented summary statistics and present forest plots with I^2^ and p values to describe heterogeneity. We found that all included studies suffered from high rates of attrition between pharmacy screening and follow up. We have also identified a strong trend towards higher rates for referral in more recent studies.

**Conclusions:**

Our results show that pharmacies are feasible sites for screening for diabetes and those at risk of cardiovascular disease. A significant number of previously unknown cases of cardiovascular disease risk factors such as hypertension, hypercholesterolemia and diabetes are identified, however a significant number of referred participants at high risk do not attend their practitioner for follow up. Research priorities should include methods of increasing uptake to follow up testing and early intervention, to maximise the efficacy of screening interventions based in community pharmacies.

## Introduction

Globally, the prevalence of type 2 diabetes (T2DM) and risk factors for cardiovascular disease (CVD) have seen an upward trend in recent years [1]. Although independent conditions, these diseases can be classified as ‘lifestyle’ related diseases as they share a number of common modifiable risk factors such as obesity, hypertension and low physical activity level. Prevention, diagnosis and treatment of these two diseases require approaches which take into consideration the overlap in risk factors. It was estimated that in 2011, 366 million people were living with diabetes worldwide [2]. More worrying still is that the incidence of diabetes is increasing dramatically and 50% of people living with the condition are currently undiagnosed [2]. Conservative estimates suggest that diabetes accounts for a total worldwide healthcare expenditure of 465 billion dollars [2] increasing to 561 billion by 2030 [3].

In 2008 CVD was the primary cause of 17.3 million deaths worldwide and like diabetes, this is expected to rise dramatically to 23.6 million by 2030 [4].

The increasing prevalence of T2DM and CVD has seen increased healthcare expenditure focussing on disease detection and early intervention to delay progression and the onset of complications. In both the United Kingdom (UK) and in the United States (US), guidance has been introduced to encourage vascular risk assessment including T2DM risk in adults aged 40 and above. Economic evaluation has shown that screening for T2DM is cost effective and may be cost saving from a health system perspective [5]. It is estimated that population based screening for cardiovascular disease in the UK alone using a simple risk score incorporating routine data in 60% of the population could prevent up to 26,789 events annually [6].

The vast majority of research in this area thus far has focussed on practice based screening to identify high risk patients to invite for testing. A number of simple risk assessment tools have been developed to pre-screen large numbers of individuals and target high risk individuals with invasive blood tests [7]. Although successful in detecting cases of undiagnosed diabetes, screening at locations such as GP surgeries could have the potential to widen health inequalities. Current screening interventions offered through GP's are associated with lower levels of uptake in BME groups and those from lower socioeconomic groups who are known to suffer from higher rates of lifestyle related diseases such as diabetes and CVD [8].

Community pharmacists are already actively involved in the management of T2DM and CHD and their involvement has shown beneficial effects in patient education and disease management [9], [10] [11]. In the context of health screening, pharmacists are known to be knowledgeable specialists but seen as an underused resource within the primary care health team [12]. Community pharmacists are estimated to have face to face contact with around 90% of the population annually [12]. Health screening based within the pharmacy and out in the community represents a valuable opportunity to potentially engage with groups who may be less likely to access GP based healthcare or be empowered for self-care including the elderly, those from lower socio-economic backgrounds or from minority ethnic groups [1]. Potential increased uptake in hard to reach groups has been demonstrated by one previous UK based programme which found high levels of participation in both males and black and minority ethnic groups (BME) groups [13]

Pharmacists are ideally placed to support existing screening methods by signposting customers to other services run by pharmacy staff for example smoking cessation. Smoking is cited as one factor in reduced health outcomes in groups with higher deprivation [14].

Almost a decade ago the paucity of research in the area of pharmacist initiated disease detection and case finding was identified by a review of the literature [11]. Although there have been a small number of studies evaluating opportunistic methods of pharmacy screening for chronic disease globally. Thus far, there has been no synthesising of this data and no evaluation of the overall success of past screening interventions worldwide.

The purpose of this systematic review is to evaluate current literature focussing on pharmacy based screening interventions for T2DM and CVD. We will evaluate response rates to pharmacy based screening as well as numbers of people either diagnosed or defined as ‘high risk’ by a pharmacy risk assessment or screening test in order to quantify the level of success of opportunistic pharmacy led screening interventions to better inform the design and delivery of future services.

## Methods

### Data Sources and Searches

We searched the Cochrane central register of controlled trials, MEDLINE and EMBASE databases from 1950 until April 2012. The search strategy comprised of four layers of search terms relating to T2DM, CVD, pharmacy and screening programmes. Keywords and medical subject headings were used to identify papers reporting uptake or yield of screening programmes, with the first phase of screening taking place at pharmacies. No language restrictions were used in the selection of papers. An example of the review protocol and electronic search strategy used can be found in the online appendix. Studies were reviewed at the title, abstract and full text stage by two independent reviewers (AW and PR), disagreements were resolved through discussion and third party advice from other co-authors was sought where necessary. Authors from the selected full texts were contacted by post and email to provide any missing data relating to the main outcomes considered.

### Study Selection

We included studies screening people for either T2DM or CVD, whereby the first contact made between the participant and healthcare professional was in a community pharmacy. We defined CVD screening as either calculation of CVD risk based on a validated scoring algorithm or measurement of blood pressure, lipids or triglyceride levels.

T2DM screening was defined as calculation of diabetes risk based on a validated scoring algorithm or assessment of known risk factors or measurement by a pharmacist of; blood or plasma glucose (either fasting or non-fasting), HbA1c, or any combination of the aforementioned methods.

### Data Extraction and Quality Assessment

Risk of bias was assessed independently by two reviewers using the US Preventive Services Task Force (USPSTF) Quality Rating Criteria [15]. The process involves evaluating each study based on a number of characteristics including; blinding, drop out, measuring procedures used and appropriate statistical analysis techniques and grading as ‘good’, ‘fair’, or ‘poor’.

### Data Synthesis and Analysis

Two main outcomes were assessed namely i) referral rate to primary care and ii) the uptake to the primary care referral. The referral rate was defined as the number referred divided by the number screened. Uptake was defined as the number attending their general practitioner divided by the number referred.

The log odds of referral were calculated as ln(number referred/(number screened-number referred)) with standard error √((1/number referred)+(1/number screened-number referred)). Similar formulae were used for the uptake. We also calculated pooled rates for percentage of the screened population who exceeded cut offs for hypertension, hypercholesterolemia and T2DM. The log odds were pooled using a random effects model to take into account heterogeneity between studies. Outcomes were back transformed by taking exponentials and reported as mean referral and uptake rates with 95% confidence intervals. Analysis was carried out in Stata (version 12).

## Results

### Summary Characteristics and Quality of Included Studies

**Figure 1:**
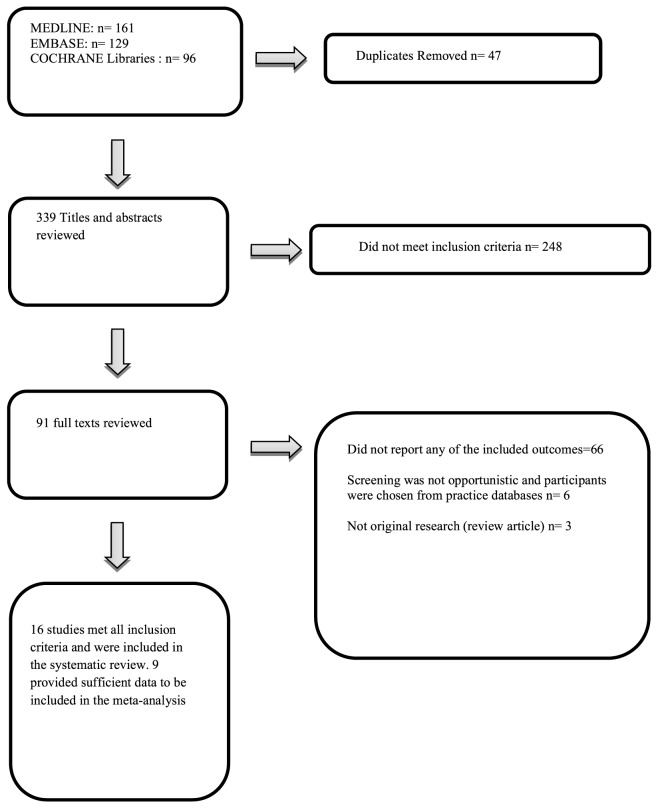
Trial Flow.

16 individual studies were included in this review [16]–[31] (see figure 1). In total, 108,414 participants were screened for CVD risk factors including cholesterol, blood pressure and T2DM. Participants screened had a mean age of 54.6 years and 56.6% were female. Seven of the studies were conducted in North America, four in the UK, three in Australia, one in Thailand and one in Switzerland. Five studies reported results following diabetes testing or diabetes risk assessment and 15 of the included studies reported results of CVD risk factor screening (see table 1). 9 studies provided data which was included in the meta analysis [17], [20], [23], [24], [26]–[30]. All 9 studies provided data on percentage of the screened population referred. One paper published by Krass et al [20] included two trial arms testing different methods of screening. The two methods had differing rates for referral and uptake of confirmatory testing and were included in the analysis separately. One paper [21] included outcome data from two sub-groups, only one of which met the inclusion criteria of an opportunistic method of recruitment. As a result we have excluded participants recruited through a postal invitation from this study. Five studies provided data on uptake of a referral to their general practitioner [20], [23], [24], [26], [28], [30].

**Table 1:** Summary of Included Studies.

First Author	Year	Quality Rating	Country	Number Screened	%men	%women	Mean Age (years)(SD)	Total % referred	Uptake	Exceeded Diabetes cut off	Exceeded BP cut-off	Exceeded Cholesterol cut off
**Donyai ** **[^28^]**	2009	Fair	UK	8287	35.5%	64.5%	NR	NR	NR	NR	NR	NR
**Earle ** **[^26^]**	2001	Fair	UK	263	43.7%	56.3%	54.1	16	NR	NR	46.8%‡	NR
**Edwards ** **[^27^]**	1981	Poor	UK	215	44.0%	56%	NR	6.1%	92.3%	NR	6.0%‡	NR
**Hersberger ** **[^25^]**	2006	Good	Switzerland	93,258	33.1%	66.9%	60.9 (14.1)	9.0%	12.8%	6.9%‡‡	14.2%*	NR
**Horgan ** **[^24^]**	2009	Good	UK	1141	60.0%	40%	NR	70.1%	NR	3.0%††	32.4%*	29.8%***
**Hourihan ** **[^23^]**	2003	Good	Australia	204	29.0%	71%	44 (13)	29.9%	NR	NR	17.7%*	17.7%***
**Jafari ** **[^22^]**	2001	Good	USA	301	65.0%	35%	57	NR	NR	NR	NR	49.8%§§
**Karwalajtys ** **[^21^]**	2009	Good	Canada	317	40.9%	59.1%	70.9 (10.8)	55.8%	43.9%	NR	55.8%*	NR
**Krass(a) ** **[^17^]**	2007	Poor	Australia	802	26%	74%	NR	28%	56.4%	28.1%¶	NR	NR
**Krass(b) ** **[^17^]**	2007	Poor	Australia	484	36%	64%	NR	24.2%	42.7%	24.4%§	NR	NR
**Peterson ** **[^20^]**	2010	Good	Australia	640	28.6%	71.4%	NR	73%	82.7%	5.5%**	30.0%*	40.0%***
**Pongwecharak ** **[^19^]**	2010	Good	Thailand	350	NR	NR	48.6 (9.9)	NR	NR	NR	29.4%*	NR
**Gardner ** **[^18^]**	1994	Good	USA	97	63.9	36.1	48.0 (18)	NR	NR	NR	17.5%†	48.4%§§
**Olenak ** **[^13^]**	2010	Fair	USA	239	28	72	NR	NR	41%	30.5%**	NR	NR
**Madjeski ** **[^16^]**	1996	Good	USA	539	35.0%	65%	NR	NR	48%	NR	NR	77.9%§§
**Hutchinson ** **[^15^]**	1979	Fair	USA	926	NR	NR	NR	NR	NR	NR	8.1%‡	NR
**Mangum ** **[^14^]**	2003	Good	USA	351	NR	NR	63	34.5%	NR	NR	13.4%*	NR

NR = Not reported.

(a) & (b) refers to two study arms from the same two arm randomised study reporting differing rates for the two main included outcomes.

**Cut-offs Used.**

**Diabetes.**

§ = tick test scoring method (more than 1 recognized risk factor for diabetes) and fasting blood glucose ≥5.5 mmol/l or random blood glucose ≥11 mmol/l.

¶ = tick test scoring method (more than 1 recognised risk factor for diabetes).

** = Fasting blood glucose ≥8 mmol/l,

†† = random blood glucose ≥10 mmol/l,

‡‡ = random blood glucose or ≥11 mmol/l fasting blood glucose 6 mmol/l.

**Blood Pressure.**

* = ≥160/100 mmHg,

† = ≥160 mmHg systolic only,

‡ = ≥140/100 mmHg.

**Cholesterol.**

§§ = total cholesterol ≥200 mg/dl,

*** = total cholesterol ≥232 mg/dl.

### Overview of screening interventions

All except two of the studies were observational [20], [22]; both were trials with some degree of randomisation between screening methods. Five of the studies integrated a sequential screening strategy into the study design with the first stage of the screening process being a non-invasive test. In the majority of cases this was done using a risk score or comparison against pre-selected risk factor cut offs based on age, ethnicity or body mass index. All of the included studies carried out the majority of screening appointments in a pharmacy setting. The majority of screening was performed in a community pharmacy setting. Only one study included a small sub-sample screened during an outreach screening session in a local elderly housing facility [17]. Of the four studies that provided data, mean consultation time was 10 minutes 30 seconds. Generally, the method in which participants found to be at risk were referred to their clinician was poorly reported. The most common form of referral in studies that did provided data used a print out of their screening results and advised high risk patients to visit their practitioner. Only four of the included studies provided the clinician with a copy of the results by post or by fax.

### Risk of Bias Assessment

Eleven studies were graded as good [17], [19], [21]–[28], [31], three studies fair [16], [18] [29] and two studies poor [20], [30] using the US Preventive Services Task Force (USPSTF) Quality Rating Criteria [15]. The most common reason for studies being graded as either fair or poor was the quality in describing the screening intervention. Exactly who carried out the consultation in addition to the contact time was particularly poorly reported. The study graded as poor had a high rate of drop out and high levels of missing data.

### Percentage of screening population referred and uptake of referral

Significant heterogeneity was found for both main outcomes (p = <0.001), we have therefore presented forest plots showing the two main outcomes reported by the included studies with 95% confidence intervals. We have not presented the calculated summary statistics due to the significant heterogeneity. This is in accordance with previously published guidance [32].

**Figure 2:**
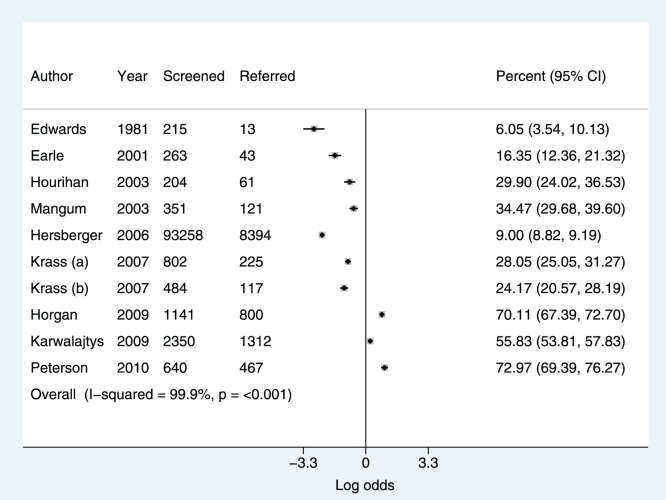
Percentage of screening population referred to their practitioner.

**Figure 3:**
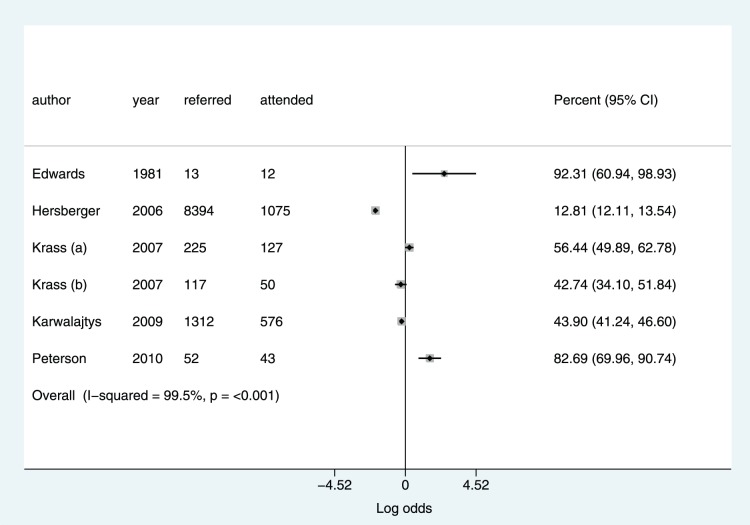
Percentage uptake of a referral to a general practitioner.

Figure 2 displays percentages of the study population referred to their practitioner. There was a strong trend towards higher referral rates in more recent studies. Figure 3 percentages of the referred population who attended their practitioner. The I^2^ statistic showed statistically significant heterogeneity for both outcomes with I^2^ greater than 75% in all analyses.

**Figure 4:**
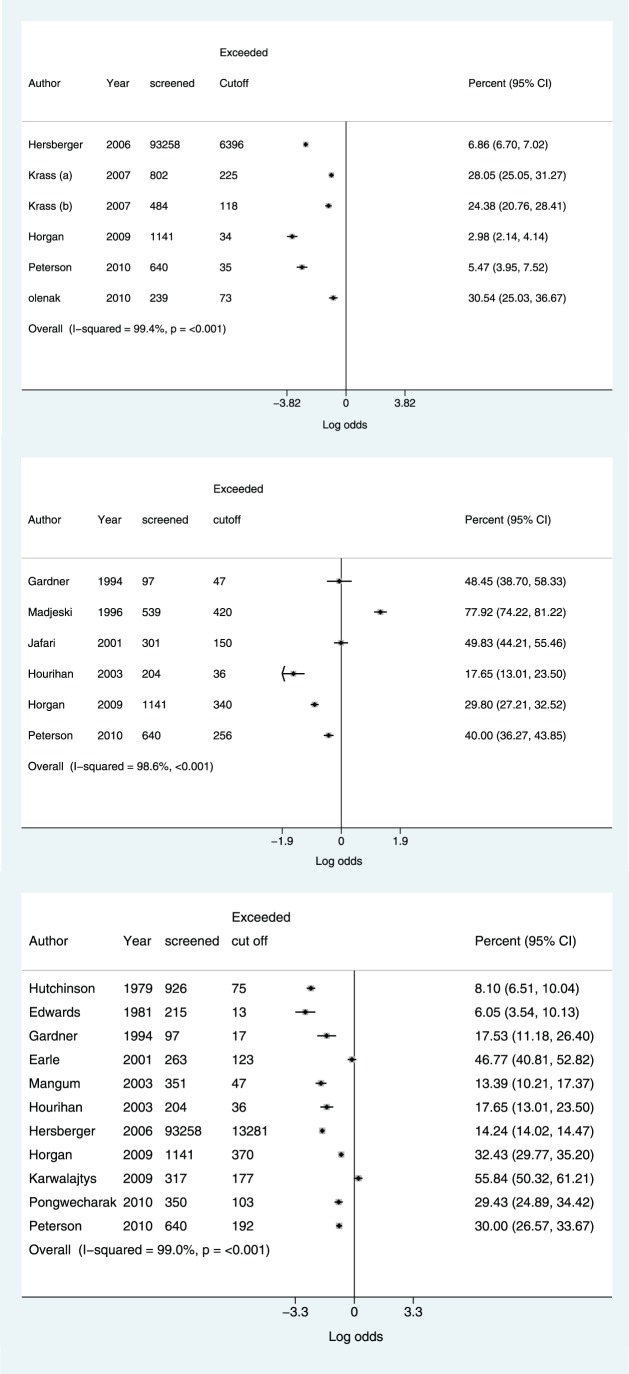
Percentage of individuals exceeding cut offs by risk factor. i. Diabetes. ii. Hypercholesterolemia. iii. Hypertension.

The percentages of individuals who exceeded diagnostic cut offs for hypertension, hypercholesterolemia and T2DM during a pharmacy based test are shown in figure 4 (i,ii and iii).

Referral cut points for CVD risk factors varied slightly between studies. A blood pressure cut point of ≥140/90 mmHg was used by the majority of studies [17], [22]–[24], [26], [27]. One study used a cut point based on systolic pressure of ≥160 mmHg [21]. Four studies referred participants exceeding a cut point of 140/90 mmHg [18], [28], [29], [33]. Diabetes cut points of ≥5.5 mmol/L for FBG and ≥11 mmol for RBG were used by one study [20]. This was in accordance with national guidance from the country in which the study took place [34]. A higher cut point of 8 mmol/l for FBG was used by Olenak et al as a threshold for referral.

Cholesterol cut points used were similar between studies, 3 of the included studies used a cut point for total cholesterol of 200 mg/dl [19], [25] [21]. Two studies used total cholesterol cut points of 232 mg/dl [23], [26].

### Prevalence of undiagnosed risk factors for cardiovascular disease

Follow up data from GP confirmatory testing was not routinely reported. Only one study reported data on prevalence of T2DM, impaired glucose tolerance or impaired fasting glucose defined by WHO diagnostic criteria. Krass et al report 2.1% of the screened population subsequently diagnosed as having either Impaired glucose regulation or impaired fasting glucose based on a fasting or random blood test followed by confirmatory oral glucose tolerance test. The same study reported screen detected prevalence rates of 0.2% and 1.7% from the two trial arms [20]. A screen detected prevalence of previously undiagnosed high total cholesterol of 17.28% was reported by one study Jafari et al [25]. Only one study reported prevalence of undiagnosed hypertension. A prevalence of 6% was reported by Mangum et al [17].

## Discussion

Overall, our analysis and results show that typically, less than half of people who take part in studies based on opportunistic recruitment to pharmacy screening for cardiovascular disease risk factors are referred to their general practitioner for a follow up appointment. A significant proportion are not followed up or do not attend their general practitioner.

We found evidence of a strong trend towards higher rates of referral in more recently published studies. There was a very high level of heterogeneity for both of these outcomes with values for referral rate ranging from 6.05%–73.13% and values for percentage take up of this referral ranging from 12.81–83.12%. This heterogeneity could have been caused by a number of factors. It is likely that different methods of measurement of uptake to referral accounted for a significant proportion of the variability.

From a health economics perspective higher drop-out rates could increase the cost per case detected from screening interventions [35]. By reducing this drop out a higher screen detected prevalence would be expected, thus reducing the cost per case detected.

The rates reported for the percentage of individuals exceeding diagnostic criteria for: hypertension, hypercholesterolemia and/or diabetes from pharmacy based screening interventions are typically higher than rates for overall diagnosed prevalence of these risk factors. Prevalence of CVD risk factors amongst pharmacy customers is likely to be higher than the general population as a majority will be attending to collect medication for a condition. Data from a UK study [27] included in our analysis showed that baseline values for CVD risk factors such as BMI and blood pressure were all higher in pharmacy customers than in the general population [36].

It is difficult to compare data on referral and uptake with findings from previous literature. Response rates to a postal invitation to a GP based screening programmes are generally high [8]. Due to the nature of opportunistic recruitment it is difficult to collect comparable data. Maximising this uptake to pharmacy screening is still of importance however, it may be possible for future screening programmes to collect data that gives an indication of the actual uptake so that this may be compared to other methods of screening.

Comparison is possible between pharmacy and GP initiated screening when considering the percentage of screened participants attending a follow up test. One previous GP initiated T2DM screening intervention reported a 94% uptake of confirmatory testing and 70% of participants completing the screening overall [8]. The substantially lower follow up rates from pharmacy initiated screening are likely to be a symptom of inadequate referral methods between pharmacists and GPs. Development of working relationships between pharmacists and GPS, together with more robust referral methods are necessary to ensure the appropriate follow up of participants identified as high risk by pharmacy based screening.

The finding that more recent studies reported a higher percentage of referrals following a screening appointment is perhaps not surprising. The rising global prevalence of CVD risk factors such as obesity [37], hypertension, hypercholesterolemia [38] and diabetes [39] would logically lead to a higher number of individuals from a screening population crossing referral thresholds for blood pressure, cholesterol or blood glucose resulting in a larger number of referrals. Increased focus in recent years on the prevention in addition to treatment of lifestyle related diseases has seen the identification of clearly defined pre-diabetic states known as impaired glucose regulation and impaired glucose tolerance. Because of this more participants may be referred with a suspected ‘high risk status’ in addition to being suspected of being undiagnosed with a CVD risk factor.

### Strengths/Weaknesses

The main strength of our study was the use of robust search, review and meta-analysis methods to provide an assessment of the past level of success of previous pharmacy initiated screening interventions. We have also identified a key weakness in past screening interventions which be given greater consideration in the design of future studies.

The main weakness of this review and meta-analysis was due to the heterogeneity in selected outcomes. As a result of this, we were unable to calculate and present summary statistics. Research in the area of community pharmacy is sparse, poorly reported and typically of relatively poor methodological quality. It is possible with an increased number of screening interventions in the future which are well evaluated and properly reported; future meta-analysis may have more success in calculating pooled rates which may be of greater use in informing the planning of future interventions.

One other potential weakness in our analysis results from the way in which the outcomes included in the meta-analysis were measured. In general, the included papers were of sound methodological quality; however, both of our main outcomes were themselves not major outcomes in any of the included studies. Subsequently there was variation in the method of measurement used. Preferred method of reporting for this outcome was through direct access to practice based medical records following a pharmacy referral. This method was reported in only two of the included studies [20], [24]. Four studies measured referral rates via a questionnaire with three of those questionnaires being filled out by the research participants [16], [19], [23] and one being filled out by the practitioners to whom the referrals were made. Response rates for these questionnaires varied and were lowest for the practitioner questionnaires (12.8%) and it is likely that such low response rates would lead to significant selection bias in such studies. It could be hypothesised that referred participants who do not attend a referral may be likely to return a questionnaire; percentage uptake of referrals would therefore be higher amongst a sample of participants that did return follow up questionnaires. As a result it is important to consider that the results gained by such questionnaires only apply to the sub group who returned the questionnaires and not necessarily the total population screened.

The results of this study highlight a need for improvement in the implementation of opportunistic pharmacy based screening programmes in order to minimise the drop out of referred patients. The level of drop out from screening programmes for T2DM and CVD risk factors represents a significant waste of investment. Screening interventions delivered by community pharmacists have the potential to increase ease of access to screening in order to reduce health inequalities particularly in the area of T2DM and CVD.

## Conclusion

The findings of this review show that previous studies of opportunistic pharmacy based screening interventions have been successful in identifying a significant proportion of the population, both suffering from and at high risk of CVD orT2DM. We have shown that more recent screening strategies have identified a higher number of high risk individuals referred to their practitioner for follow up. However the review has also shown that a high proportion of those individuals found to be at high risk of CVD or T2DM do not attend a follow up appointment with their practitioner.

It is vital that future screening interventions are designed to minimise this drop out in order to maximise both the financial and health related gains from increased investment and interest in future screening interventions in pharmacies worldwide.

## Supporting Information

Checklist S1 **PRISMA Checklist.**
(DOC)
